# Divergent Ventilatory and Blood Pressure Responses are Evident Following Repeated Daily Exposure to Mild Intermittent Hypoxia in Males with OSA and Hypertension

**DOI:** 10.3389/fphys.2022.897978

**Published:** 2022-05-26

**Authors:** Gino S. Panza, Shipra Puri, Ho-Sheng Lin, Jason H. Mateika

**Affiliations:** ^1^ John D. Dingell Veterans Affairs Medical Center, Detroit, MI, United States; ^2^ Department of Physiology, Wayne State University School of Medicine, Detroit, MI, United States; ^3^ Department of Otolaryngology, Wayne State University School of Medicine, Detroit, MI, United States; ^4^ Department of Internal Medicine, Wayne State University School of Medicine, Detroit, MI, United States

**Keywords:** mild intermittent hypoxia, obstructive sleep apnea, hypertension, long-term facilitation, progressive augmentation

## Abstract

**Introduction:** Resting minute ventilation and ventilation during and following hypoxia may be enhanced following daily exposure to mild intermittent hypoxia (MIH). In contrast, resting systolic blood pressure (SBP) is reduced following daily exposure to MIH. However, it is presently unknown if the reduction in resting SBP following daily exposure, is coupled with reduced SBP responses during and after acute exposure to MIH.

**Methods:** Participants with obstructive sleep apnea (OSA) and hypertension (*n* = 10) were exposed to twelve 2-min bouts of MIH (oxygen saturation—87%)/day for 15 days. A control group (*n* = 6) was exposed to a sham protocol during which compressed air (i.e., F_I_O_2_ = 0.21) was inspired in place of MIH.

**Results:** The hypoxic ventilatory response (HVR) and hypoxic systolic blood pressure response (HSBP) increased from the first to the last hypoxic episode on the initial (HVR: 0.08 ± 0.02 vs. 0.13 ± 0.02 L/min/mmHg, *p* = 0.03; HSBP: 0.13 ± 0.04 vs. 0.37 ± 0.06 mmHg/mmHg, *p* < 0.001) and final (HVR: 0.10 ± 0.01 vs. 0.15 ± 0.03 L/min/mmHg, *p* = 0.03; HSBP: 0.16 ± 0.03 vs. 0.41 ± 0.34 mmHg/mmHg, *p* < 0.001) day. The magnitude of the increase was not different between days (*p* ≥ 0.83). Following exposure to MIH, minute ventilation and SBP was elevated compared to baseline on the initial (MV: 16.70 ± 1.10 vs. 14.20 ± 0.28 L/min, *p* = 0.01; SBP: 167.26 ± 4.43 vs. 151.13 ± 4.56 mmHg, *p* < 0.001) and final (MV: 17.90 ± 1.25 vs. 15.40 ± 0.77 L/min, *p* = 0.01; SBP: 156.24 ± 3.42 vs. 137.18 ± 4.17 mmHg, *p* < 0.001) day. The magnitude of the increases was similar on both days (MV: 3.68 ± 1.69 vs. 3.22 ± 1.27 L/min, SBP: 14.83 ± 2.64 vs. 14.28 ± 1.66 mmHg, *p* ≥ 0.414). Despite these similarities, blood pressure at baseline and at other time points during the MIH protocol was reduced on the final compared to the initial day (*p* ≤ 0.005).

**Conclusion:** The ventilatory and blood pressure responses during and following acute MIH were similar on the initial and final day of exposure. Alternatively, blood pressure was down regulated, while ventilation was similar at all time points (i.e., baseline, during and following MIH) after daily exposure to MIH.

## 1 Introduction

Mild intermittent hypoxia (MIH) is defined by exposure to a few episodes of hypoxia (i.e., no greater than 15 episodes) that are short (i.e., no greater than 4 min) and accompanied by a decrease in oxygen saturation of no less than 85% (Puri et al., 2021). Acute exposure to MIH may result in the initiation of two forms of respiratory plasticity in humans. Progressive augmentation of the hypoxic ventilatory response (PA) and long-term facilitation (LTF) of ventilation (Puri et al., 2021). Progressive augmentation is characterized by a progressive increase in the ventilatory response to hypoxia from the initial to the final hypoxic episode of an MIH protocol. Long-term facilitation is characterized by an elevation in ventilation after exposure to MIH compared to baseline (Mateika and Narwani, 2009). Progressive augmentation has been observed during exposure to MIH in some studies (Harris et al., 2006; Lee et al., 2009; Gerst et al., 2011) but not others (Mateika et al., 2004; Khodadadeh et al., 2006), while LTF has been observed in healthy humans (Harris et al., 2006; Wadhwa et al., 2008; Lee et al., 2009; Vermeulen et al., 2020), humans with spinal cord injury (Tester et al., 2014) and humans with obstructive sleep apnea (OSA) (Lee et al., 2009; Gerst et al., 2011; Syed et al., 2013).

Although respiratory plasticity has been documented in humans with sleep apnea, no studies to our knowledge have reported simultaneous ventilatory and cardiovascular responses under conditions of acute MIH in this population. Thus, the first aim of our study was to document concurrent ventilatory and blood pressure responses to acute MIH in participants with sleep apnea and concurrent hypertension. We hypothesized that the ventilatory and blood pressure response to MIH would progressively increase from the initial to the final episode of a MIH protocol. We also hypothesized that ventilation and blood pressure following exposure to MIH would be greater than baseline. Overall, our findings could lend support to previous hypotheses that acute exposure to MIH leads to enhanced respiratory and cardiovascular responses to hypoxia and elevated resting levels of ventilation and blood pressure that persist for a short period of time (i.e., 30 min) following hypoxic exposure.

In addition to exploring ventilatory and cardiovascular responses to acute MIH, we were interested in documenting the simultaneous response of these variables to daily exposure to MIH. Our laboratory previously established that repeated daily exposure (i.e., 10 days) to MIH augments the hypoxic ventilatory response and the magnitude of long-term facilitation (Yokhana et al., 2012). The increase in magnitude of LTF may manifest as an increase in baseline ventilation on the final compared to the initial day. Likewise, this form of plasticity may manifest as an increase in minute ventilation after exposure to MIH, on the final day compared to the initial day of the protocol. However, it is not known if similar cardiovascular responses are evident following repeated daily exposure. Indeed, we recently showed that repeated daily exposure to MIH resulted in reductions in resting blood pressure (Panza et al., 2022). Given this finding, we hypothesized that an uncoupling of the respiratory and cardiovascular responses occurs during and following repeated daily exposure to MIH. In summary, our primary hypotheses were that repeated daily exposure to MIH would result in an augmented hypoxic ventilatory response and long-term facilitation of ventilation. In contrast, we hypothesized that systolic blood pressure would be reduced during and following repeated daily exposure to MIH.

## 2 Materials and Methods

### 2.1 Protocol

The Institutional Review Board of Wayne State University School of Medicine and John D. Dingell Veterans Affairs Medical Center approved the protocol (#030617M1FV) that conformed to the standards set by the Declaration of Helsinki and is registered in the Clinical Trials data base (#NCT03736382). All participants provided written consent prior to enrolling in the study. The sample size calculation has been reported elsewhere (Panza et al., 2022). The calculation was completed using the primary outcome variable systolic blood pressure.

Participants were recruited from a Department of Veterans Affairs data base and from the local Detroit metro area using advertisements posted on various social media websites. One hundred individuals were assessed for eligibility. Seventy-five individuals did not meet the inclusion criteria (see below for criteria). The individuals that met the inclusion criteria were randomized. Thereafter, nine individuals did not complete the protocol either because of issues related to time commitment or adherence to the protocol. Sixteen male participants completed the protocol (experimental group *n* = 10, control group *n* = 6). A flow diagram detailing recruitment of these participants has been previously published (Panza et al., 2022).

A schematic depiction of the study design has been previously published (see Figure E1 in online supplement Panza et al., 2022). On Day 1 participants were screened to confirm the presence of untreated hypertension (i.e., ≥130/80 mmHg) without accompanying co-morbidities besides OSA. The inclusion criteria included participants who were not treated with medication (e.g., blood pressure medication, selective serotonin re-uptake inhibitors, metformin) or continuous positive airway pressure. Likewise, participants were included if their 1) apnea/hypopnea index was greater than five events/hr. and their sleep efficiency was greater than 75% (see day 2 below) 2) forced vital capacity was greater than 80% and their FEV_1.0_/FVC was greater than 70% of predicted values 3) body mass index was less than 40 kg/m^2^.

On day 2, an overnight polysomnogram was completed to confirm the presence of OSA. On day 3, a polysomnogram was completed to determine the therapeutic continuous positive airway pressure and to determine the critical closing pressure that induced upper airway collapsibility. On Day 4 blood pressure was measured over a 24-h period. The data from day 3 and day 4, and similar measures that were repeated on day 19 and 20, have been published elsewhere (Panza et al., 2022). On day 5 the participants were exposed to the mild intermittent hypoxia or sham protocol (Panza et al., 2022). Thereafter, the experimental group was exposed to MIH between 7–9 AM, 5 days a week (i.e., Monday–Friday), over three consecutive weeks. Similar considerations were employed when the control group was exposed to the sham protocol. On each day of the protocol both the experimental and control groups were treated nightly with in-home continuous positive airway pressure.

### 2.2 Mild Intermittent Hypoxia/Sham Protocol

**FIGURE 1:**
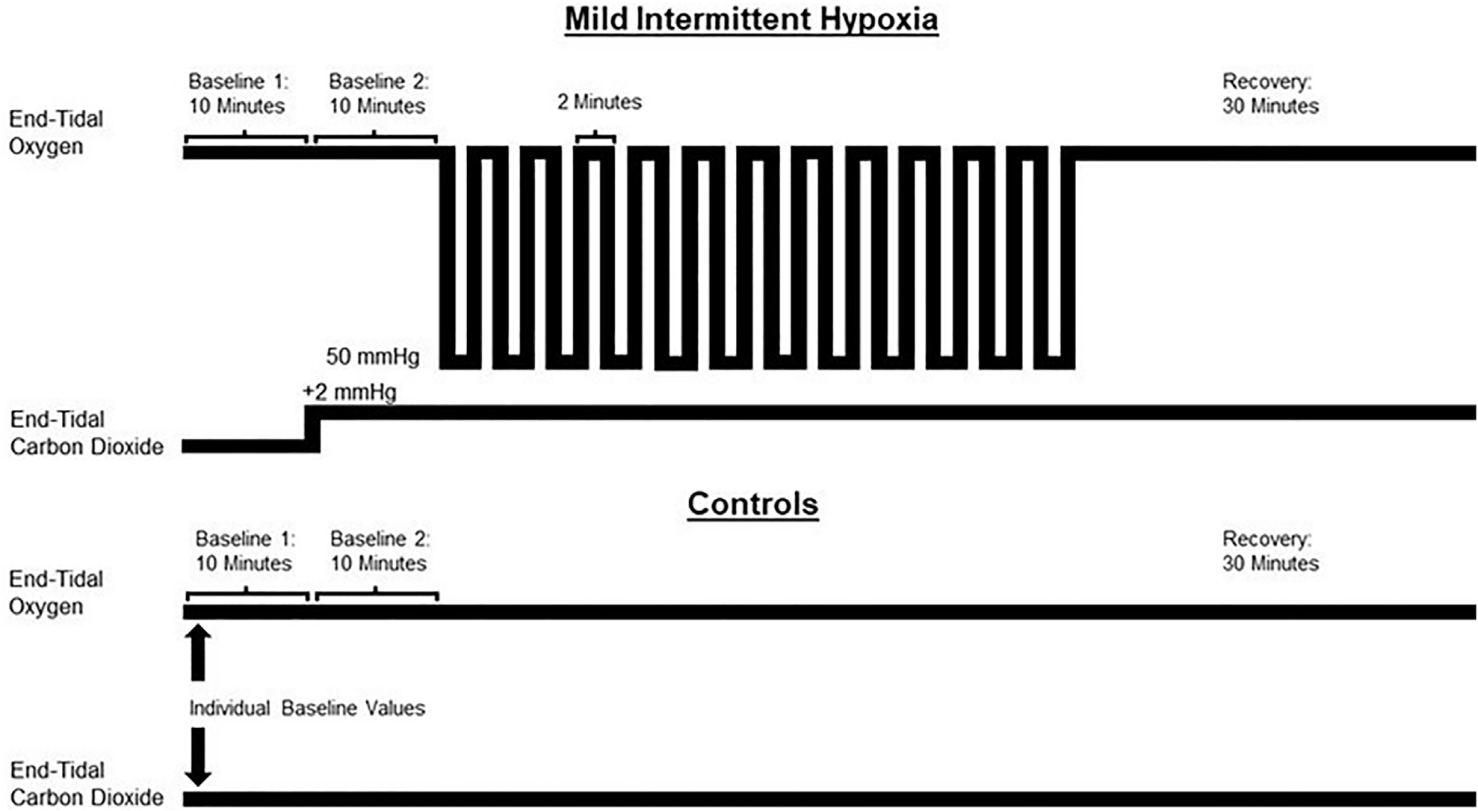
A schematic depiction of the protocol used for the experimental (mild intermittent hypoxia) and control group. The control group breathed compressed air from non-diffusible bags (21% oxygen) during the equivalent episodes in which the experimental group breathed hypoxic gas (8% oxygen). Both groups breathed room air during the recovery phases. Baseline 1 = normoxic baseline. Baseline 2 = hypercapnic baseline (experimental group). The control group breathed room air during baseline 2.

The MIH or sham protocol consisted of two 10-min baseline periods (B_1_ and B_2_, respectively) followed by 12 2-min intervals of MIH or compressed air interspersed with 2-min of breathing room air (Figure 1). During the initial baseline period, participants breathed room air for 10 min to establish baseline measures of beat to beat blood pressure, ventilation, tidal volume, breathing frequency, heart rate, oxygen saturation, and end-tidal oxygen and carbon dioxide. The next 10-min baseline period was used to establish the same parameters. However, during this period, supplemental carbon dioxide was used to maintain an end-tidal carbon dioxide of 2 mmHg (i.e., mean = 2.06 ± 0.07 mmHg) above baseline levels in the experimental group. This level of carbon dioxide was then maintained throughout the remainder of the protocol. At the conclusion of the second baseline (B_2_), the participants were exposed to 12 2-min episodes of hypoxia that were induced by inspiring 8% oxygen from a non-diffusible bag until the partial pressure of end-tidal oxygen was between 50–55 mmHg. This pressure corresponded to an oxygen saturation of 85%–88%. At the conclusion of each hypoxic episode, one breath of 100% oxygen was delivered to clear the lungs of the hypoxic gas. Each episode was followed by 2 min of breathing room air, with the exception of the last episode which was followed by a 30-min recovery period. 100% oxygen and 100% carbon dioxide were titrated on an as-needed basis in the experimental group to maintain appropriate end-tidal values (i.e., P_ET_O_2_ 50–55 mmHg and P_ET_CO_2_ 2 mmHg above baseline) throughout the protocol. The sham protocol mimicked the hypoxic protocol, but participants inspired compressed air (21% oxygen) from a non-diffusible bag and room air during the recovery intervals. Participants were blinded to their group allocation.

During completion of the MIH protocol, participants breathed while wearing a face mask that allowed end-tidal oxygen (model 17518, Vacumed, Ventura, CA, United States) and carbon dioxide (model 17515, Vacumed, Ventura, CA, United States) to be sampled from two separate mask ports. The face mask was connected to a pneumotachograph (model RSS100-HR, Hans Rudolph, Kansas, MO, United States), which monitored breath-by-breath changes in ventilation. The pneumotachograph was attached to a two-way valve. The inspiratory port of the valve was connected to a stopcock. Subjects inspired either room air or the contents from one of two bags attached to the stopcock that contained either 8% oxygen/balance nitrogen, or 100% oxygen. The output from a flowmeter was attached to the stopcock port connected to the inspiratory port of the valve. Gas from two cylinders containing 100% oxygen and 100% carbon dioxide were connected to the flowmeter. Thus, supplemental oxygen and carbon dioxide could be added to the 8% oxygen/balance nitrogen to maintain desired levels of P_ET_O_2_ (i.e., 50 mmHg) and P_ET_CO_2_ (i.e., 2 mmHg above baseline values).

### 2.3 Data Collection

#### 2.3.1 Polysomnography

During the diagnostic sleep study (i.e., Day 2) electroencephalogram (C3/A2, C4/A1, O1/A2, and O2/A1), electrooculograms, submental electromyography, and a three-lead electrocardiogram were recorded. Chest wall and abdominal movements were recorded using inductive plethysmography (Respitrace, Ambulatory Monitor, Inc., Ardsley, NY, United States). Airflow and tidal volume were measured using a pneumotachometer (Model RSS100-HR, Hans Rudolph Inc., Kansas, MO, United States) attached to a tight-fitting face mask. Upper airway pressure was also measured using a transducer tipped catheter (MPC-500, Millar, Inc., Houston, TX, United States). The catheter was inserted nasally until the tip of the catheter was above the base of the tongue but below the uvula. Heart rate and oxygen saturation were recorded using a pulse oximeter (BIOX 3700, Ohmeda Corp., Laurel, MD, United States). All variables were converted from analog to digital at a sampling frequency of 100 Hz per channel and input into a commercially available software package (Gamma v. 4.0, Astro-Med Inc., West Warwick, RI, United States). During titration of continuous positive airway pressure, the same physiological parameters outlined for the diagnostic sleep study were measured. However, participants were fitted with a nasal mask and the participant’s mouth was taped shut to ensure nasal breathing. Therapeutic pressure was determined both visually and later, objectively. Data acquisition software (Dataq, Windaq DI-720) was used in concert with the polysomnography data to record the ventilatory data during the in-lab titrations, and the data were sampled at a rate of 250 Hz per channel.

#### 2.3.2 Respiratory, Cardiovascular and Autonomic Measures

Minute ventilation, breathing frequency, inspiratory and expiratory time and tidal volume were collected on a breath-by-breath basis using commercially available software (LabVIEW, National Instruments, Austin, TX, United States) on each day of the protocol. Similarly, heart rate and oxygen saturation were monitored using a pulse oximeter, along with an electrocardiogram on each day of the protocol. Beat to beat blood pressure was measured using a Finapres on Day 5 and Day 19 of the mild intermittent or sham protocol. These days will be referred to as the initial and final day of the protocol from this point forward. The data was collected using commercially available software (LabVIEW, National Instruments, Austin, TX, United States; WinDaq, Dataq Instruments, Akron, OH, United States) at a sampling rate of 250 Hz.

### 2.4 Data Analysis

#### 2.4.1 Blood Pressure Variability

The blood pressure signal was visually inspected to ensure the absence of artifacts and anomalies. Subsequently, blood pressure measures were imported into a customized program created with LabVIEW graphical software (National Instruments, Austin, TX, United States). Once imported systolic blood pressure peaks were detected. Systolic blood pressure time series were interpolated and resampled at 10 Hz, channeled through a Hanning window function, and transformed into a power spectra using discrete Fourier transform algorithms. The power spectra were integrated, and the areas of interest quantified. Spectral components for blood pressure variability were expressed as both absolute units (ms^2^/Hz or mmHg^2^/Hz) and normalized units which was calculated as [absolute power of the components ÷ (total power−very low-frequency power)] × 100. Power in the low frequency range (LFSBP 0.04–0.15 Hz) and high-frequency range (HFSBP 0.15–0.40 Hz) was calculated to determine blood pressure variability. The HFSBP (mmHg^2^/Hz) is considered a function of cardiac parasympathetic nervous system activity to the heart (Pagani et al., 1988). The LFSBP (mmHg^2^/Hz), represents sympathetic modulation of the microcirculation without influence of the respiratory system (Pagani et al., 1988; Parati et al., 1995). HFSBP measures may be impacted by alterations in tidal volume and breathing frequency (Pagani et al., 1988; Parati et al., 1995). However, we monitored these variables to determine their impact on measures of blood pressure variability. LF measures may also be contaminated by respiratory oscillations if the respiratory rate is <10 breaths/min, which was not the case in our study.

#### 2.4.2 Respiratory, Cardiovascular and Autonomic Measures

**FIGURE 2:**
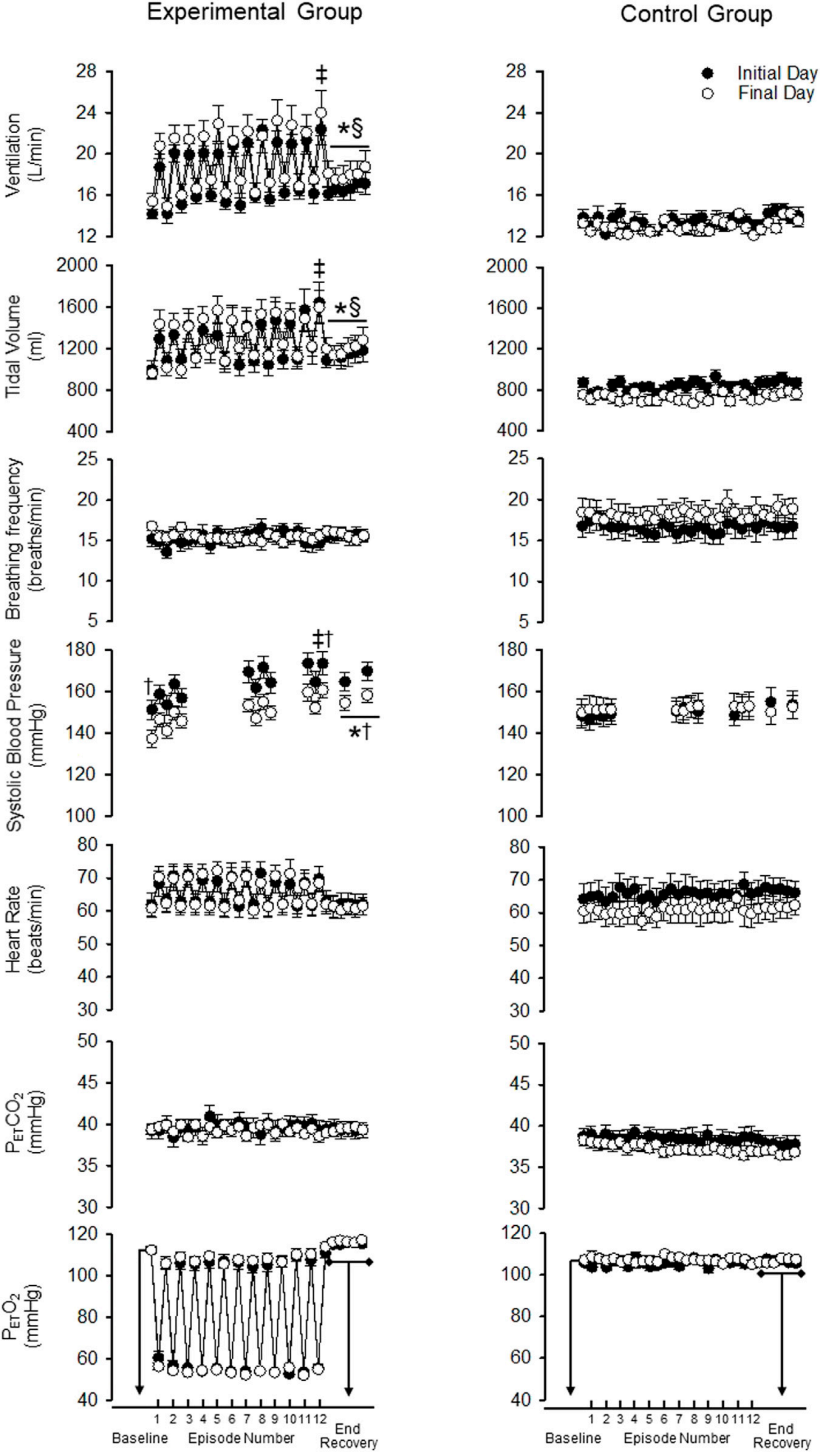
Minute ventilation, tidal volume, breathing frequency, systolic blood pressure, heart rate, end-tidal carbon dioxide and oxygen during B_2_ (i.e., baseline), each hypoxic episode (1–12, which are denoted by the tick marks on the *x*-axis), and corresponding recovery periods (1–11), as well as the 30-min end-recovery period, for both the experimental and control group. Recordings of systolic blood pressure were obtained during B_2_, hypoxic episodes 1–2, 7–8, and 11–12, recovery periods 1–2, 7–8, 11 and during two 5-min end-recovery periods that included data collected between minutes 10–15 and 25–30 of end-recovery. Note that during exposure to mild intermittent hypoxia minute ventilation and systolic blood pressure were greater during the last compared to the first hypoxia episode on a given day. Likewise, minute ventilation and systolic blood pressure were elevated during the recovery period following intermittent hypoxia compared to baseline on a given day. Lastly, note that despite these acute responses, systolic blood pressure at any given time point on the final day was less compared to the initial day even though this was not evident in the minute ventilation response. ^‡^ = significantly different than episode 1. * = significantly different compared to B_2_. ^†^ = significantly different compared to the initial day at a similar time point. ^§^ = significantly different than control at the respective time point. Statistical significance *p* < 0.05. *n* = 10 for the experimental group and *n* = 6 for the control group.

**FIGURE 3:**
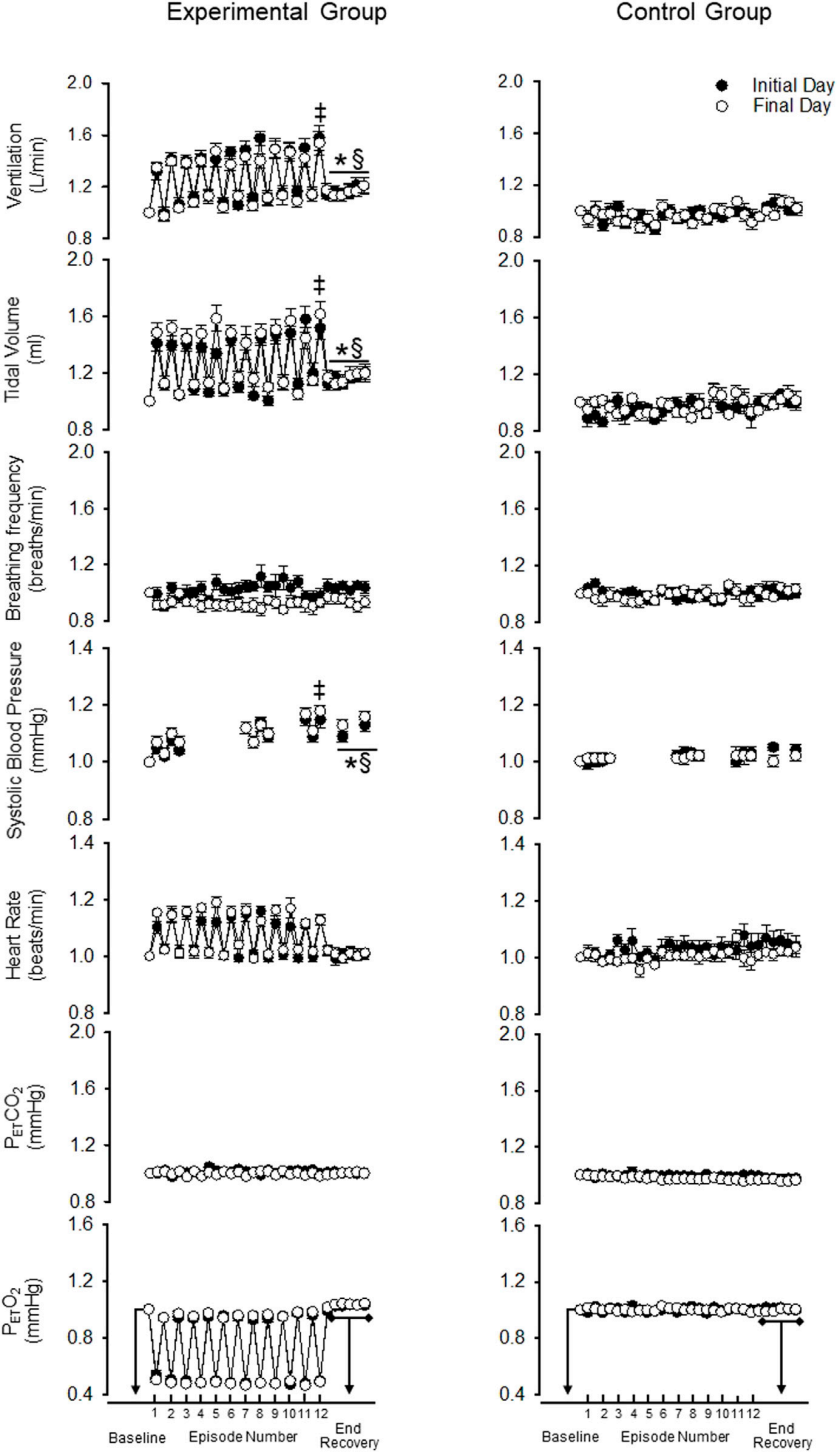
Standardized measures (standardized to baseline B_2_) of minute ventilation, tidal volume, breathing frequency, systolic blood pressure, heart rate, end-tidal carbon dioxide and oxygen during B_2_ (i.e., baseline), each hypoxic episode (1–12, which are denoted by tick marks on the *x*-axis) and corresponding recovery periods (1–11), as well as, the 30-min end-recovery period, for both the experimental and control group. Recordings of systolic blood pressure were obtained during B_2_, hypoxic episodes 1–2, 7–8, and 11–12, recovery periods 1–2, 7–8, 11 and during two 5-min end-recovery periods that included data collected between minutes 10–15 and 25–30 of end-recovery. Note that despite standardization the acute and chronic systolic blood pressure responses were similar to those described in the legend for Figure 2. ^‡^ = significantly different from the first episode. * = significantly different compared to B_2_. ^§^ = significantly different than control at the respective time point. Statistical significance *p* < 0.05. *n* = 10 for the experimental group and *n* = 6 for the control group.

Respiratory, cardiovascular and autonomic data (i.e., blood pressure variability) was averaged using the final 5 min of B_1_ (i.e., normoxia) and B_2_ (i.e., hypercapnia maintained at 2 mmHg above baseline end-tidal carbon dioxide for the MIH group, normoxia for the control group) of the MIH or sham protocol. Blood pressure and autonomic measures from B_1_ has been published elsewhere (Panza et al., 2022). Likewise, respiratory and heart rate data was averaged from the last 30 s of each hypoxic episode and recovery period, with the exception of the end recovery period, which was 30 min in length. The 30-min end-recovery period was divided into six 5-min segments as shown in Figures 1–3. However, for the completion of statistical analysis, an average value for the entire 30-min end-recovery period was used. Beat to beat systolic blood pressure was obtained for the initial two episodes (i.e., episodes 1 and 2), middle two episodes (i.e., episodes 7 and 8) and final two episodes (i.e., episodes 11 and 12), along with their corresponding recovery periods. Beat to beat blood pressure was recorded continuously between minutes 10–15 and 25–30 of end recovery (See Figures 1–3). Blood pressure and blood pressure variability measured during the end-recovery time periods (10–15 and 25–30 min) were averaged since differences did not exist between the periods. Statistical comparisons were also completed using data collected during the first and last hypoxic episode.

Administration of MIH, and the subsequent respiratory, cardiovascular and autonomic responses, on a given day are referred to as “Acute MIH” in the presentation of the results. In addition, administration of MIH, and the subsequent responses, over the 15-day period are referred to as “Daily MIH” in the presentation of the results.

#### 2.4.3 Ventilatory and Blood Pressure Response to Hypoxia

The ventilatory response and systolic blood pressure response to hypoxia was determined by subtracting the average minute ventilation or systolic blood pressure recorded during the last 5 min of B_2_ from the average minute ventilation or systolic blood pressure recorded from the last 30 s of the first or last episode of the MIH protocol. This difference was divided by the difference between the partial pressure end-tidal oxygen measured during the last 5 min of B_2_ and the partial pressure of end-tidal oxygen measured during the last 30 s of the hypoxic episode. All respiratory, cardiovascular and autonomic parameters are presented as absolute values and as a fraction of B_2_ values.

### 2.5 Statistical Analysis

4A two-tailed unpaired t-test was used to compare participant characteristics at baseline. A 2 × 2 repeated measures analysis of variance with a Student Newman-Keuls post-hoc analyses was used to determine if differences in respiratory parameters, systolic blood pressure and blood pressure variability were evident during end-recovery compared to B_2_ on the initial and final day of the MIH or sham protocol. The factors were Group × Time Point. A similar analysis was used to determine if differences in the ventilatory or systolic blood response to hypoxia were evident during the first and last hypoxic episode of the MIH protocol. If normality failed, the data were transformed (i.e., natural log). Only systolic blood pressure failed normality after transform. In this case, a repeated measures analysis of variance was completed on each group individually. Comparisons between groups at each time point was then computed using a two-tailed unpaired *t*-test. Correlations were completed using a Pearson correlation coefficient. A Cohen’s *d* effect size is also provided for minute ventilation and systolic blood pressure data. Significance was set at *p* ≤ 0.05 and *p* > 0.05 and <0.10 was considered to indicate a trend toward significance. Data is presented as mean ± standard error.

## 3 Results

### 3.1 Participant Characteristics

Sixteen male participants with untreated OSA and hypertension completed the protocol. The anthropometric and baseline characteristics of the participants are shown in Tables 1 and 2. Changes in blood pressure while breathing room air (i.e., B_1_) have been previously reported (Panza et al., 2022). Specifically, systolic and diastolic blood pressure was reduced on day 15 compared to day 1 while breathing room air (SBP: 129.71 ± 3.00 vs. 143.99 ± 2.86; DBP: 82.04 ± 3.88 vs. 90.04 ± 2.33, *p* < 0.001).

**TABLE 1:** Participant characteristics.

Variable	Experimental	Control
Age (years)	40.7 ± 3.1	46.2 ± 4.2
Height (cm)	174.2 ± 3.1	180.7 ± 2.7
Weight (kg)	98.7 ± 7.2	105.2 ± 7.3
BMI (m^2^/kg)	32.4 ± 1.4	32.3 ± 1.9
Forced vital capacity (L)	4.1 ± 0.3	3.9 ± 0.6
FEV_1.0_ (L)	3.2 ± 0.2	3.3 ± 0.5
FEV_1.0_/FVC	0.78 ± 0.02	0.84 ± 0.01
Systolic blood pressure (mmHg)	147.2 ± 2.0	152.7 ± 2.0
Diastolic blood pressure (mmHg)	88.5 ± 3.0	92.8 ± 3.1
Resting oxygen Saturation (%)	96.4 ± 0.2	96.2 ± 0.3
Baroreceptor sensitivity (ms/mmHg)	8.8 ± 1.4	9.7 ± 1.3
Ethnicity	African American (n = 4), Asian (n =1), Caucasian (n = 4), Hawaiian (n = 1)	African American (n = 6)

FEV_1.0_, Forced expiratory volume in 1 s; FVC, Forced vital capacity.

**TABLE 2:** Polysomnography characteristics.

Variable	Experimental	Control
Total sleep time (minutes)	301.4 ± 15.1	312.1 ± 29.9
Sleep efficiency (%)	78.1 ± 2.9	78.6 ± 2.9
N1 sleep (%)	35.5 ± 7.5	47.4 ± 11.1
N2 sleep (%)	51.7 ± 4.9	43.1 ± 7.3
N3 sleep (%)	7.0 ± 3.5	0.3 ± 0.3
REM sleep (%)	5.2 ± 1.5	9.2 ± 5.3
AHI (Events/Hour)	45.4 ± 5.4	44.7 ± 11.9
AI (Events/Hour)	23.8 ± 5.4	26.0 ± 11.5
HI (Events/Hour)	21.6 ± 3.5	18.7 ± 5.7
OA duration (seconds)	20.4 ± 1.4	18.5 ± 1.0
OH duration (seconds)	22.1 ± 2.6	18.8 ± 1.0
Average SaO_2_ desaturation (%)	91.5 ± 0.7	93.2 ± 0.6
Lowest SaO_2_ desaturation (%)	81.5 ± 3.1	86.1 ± 2.0
Arousal index (Arousal/Hour)	56.5 ± 9.5	63.6 ± 13.0

AHI, Apnea hypopnea Index; AI, Apnea Index; HI, Hypopnea Index; OA, Obstructive Apnea; OH, Obstructive Hypopnea; SaO_2_, Oxygen Saturation.

### 3.2 Minute Ventilation Response During and Following Exposure to Mild Intermittent Hypoxia

#### 3.2.1 Acute Mild Intermittent Hypoxia

**FIGURE 4:**
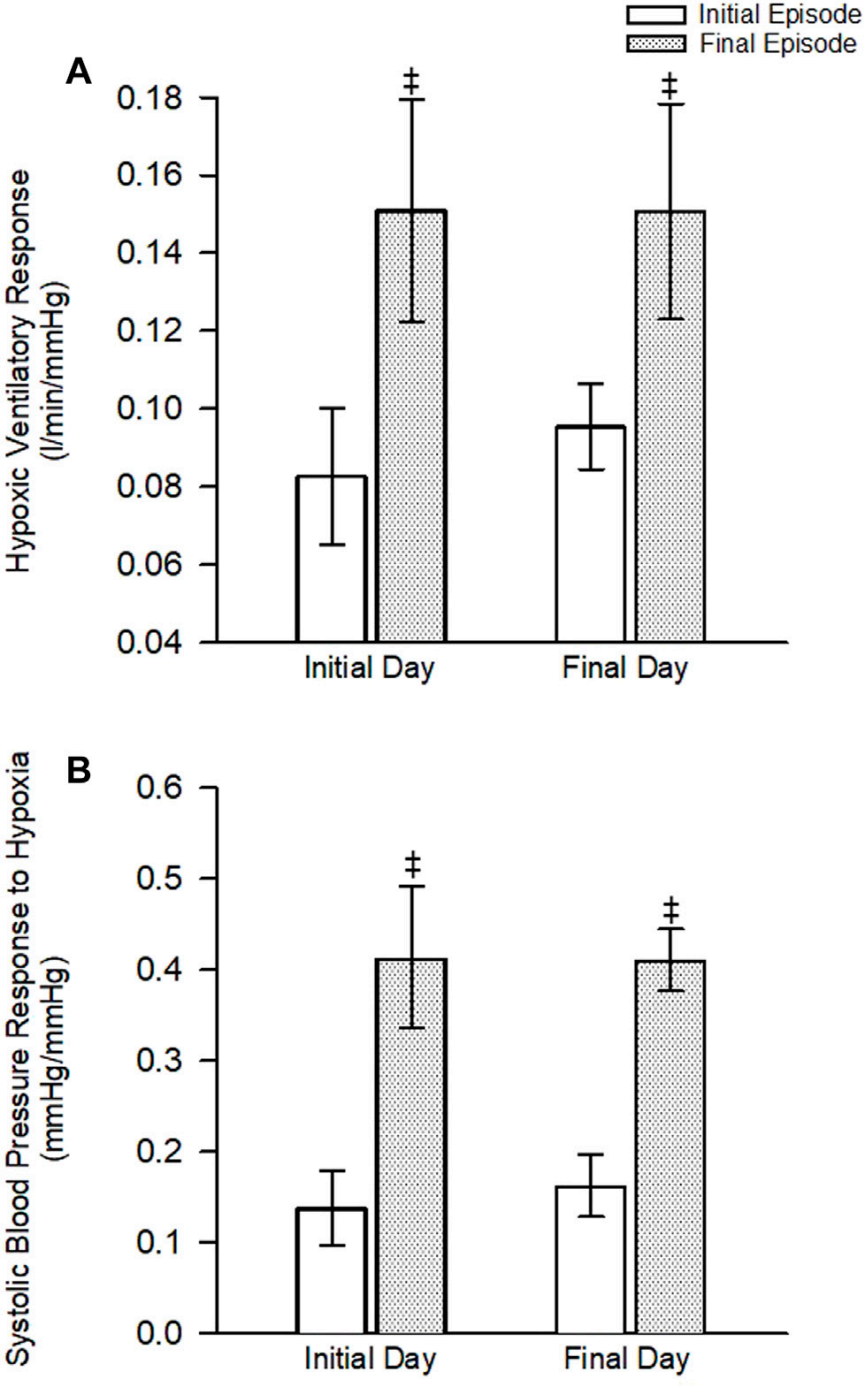
**(A)** The ventilatory response to hypoxia during the first and last hypoxic episode on the initial and final day of the protocol. **(B)** The systolic blood pressure response to hypoxia during the first and last hypoxic episode on the initial and final day of the protocol. Note that the ventilatory and systolic blood pressure response was increased during the last episode compared to the first episode on both the initial and final day of the protocol. No differences between the initial and final day were noted. ^‡^ = significantly different from the first episode. Statistical significance *p* < 0.05. *n* = 10 for the experimental group and *n* = 6 for the control group.

As expected, minute ventilation increased in response to hypoxia in the experimental group (Figures 2, 3—NB symbols indicating statistical significance not shown for this expected increase) (*p* < 0.001). A gradual increase in minute ventilation from the first to the last episode of the MIH protocol was evident on the initial (*p* ≤ 0.021, *d* = 0.86) and final (*p* ≤ 0.050, *d* = 0.60) day (Figures 2, 3). In addition, the hypoxic ventilatory response was greater during the last compared to the first episode on the initial (*d* = 0.86) and final day (*d* = 0.9) (*p* = 0.025 for both the initial and final day) (Figure 4).

Minute ventilation during end recovery (i.e., following exposure to hypoxia) was significantly greater than B_2_ on the initial (*d* = 1.25) and final day of the MIH protocol (Figure 2) (*d* = 0.81) (*p* ≤ 0.007 for both the initial and final day). This increase was also evident when the standardized data (Figure 3) (*p* < 0.001) was compared. The increase in minute ventilation was primarily the consequence of an increase in tidal volume compared to B_2_ (Figures 2, 3) (*p* ≤ 0.026), since breathing frequency during end recovery was similar to B_2_ on the initial and final days (Figures 2, 3) (*p* ≥ 0.454).

In contrast to the findings in the experimental group, minute ventilation, tidal volume and breathing frequency were similar during end-recovery compared to B_2_ in the control group on the initial (minute ventilation *d* = 0.13) and final days (minute ventilation *d* = 0.15) (Figures 2, 3) (*p* > 0.821 in all cases). Consequently, minute ventilation during end recovery on the initial and final day was greater in the experimental group compared to control (Figure 2) (*p* < 0.001). This increase was also evident when the standardized (Figure 3) (*p* < 0.001) data was compared.

#### 3.2.2 Daily Mild Intermittent Hypoxia

Minute ventilation, tidal volume and breathing frequency during B_2_ were similar on the initial and final days of the MIH protocol (Figure 2) (*p* ≥ 0.186). Likewise, the magnitude of minute ventilation (Figures 2, 3) and the hypoxic ventilatory response (Figure 4A) during the first or last episode of the MIH protocol was similar on the initial and final day (*p* ≥ 0.285—absolute minute ventilation, episode 1 *d* = 0.54, episode 12 *d* = 0.28; *p* > 0.407—hypoxic ventilatory response, episode 1 *d* = 0.35, episode 12 *d* = 0.20). Moreover, minute ventilation, tidal volume and breathing frequency during end-recovery were similar on the initial and final day (minute ventilation: *d* = 0.40) of the MIH protocol (Figures 2, 3) (*p* > 0.126). As expected, minute ventilation, tidal volume, and breathing frequency were similar on the initial and final day in the control group when absolute or standardized data were compared (*p* > 0.279 for all comparisons).

### 3.3 Heart Rate

#### 3.3.1 Acute Mild Intermittent Hypoxia

Heart rate increased during each hypoxic episode compared to B_2_ on the initial and final day of the protocol in the experimental group (Figures 2, 3) (*p* < 0.001). However, a progressive increase in the heart rate response to hypoxia from the initial to the final episodes of the MIH protocol was not evident (Figures 2, 3), on either day (*p* ≥ 0.675 in all cases). Additionally, heart rate was not different during end-recovery, compared to baseline, on the initial or final day of the protocol (Figure 2) (*p* ≥ 0.859). Likewise, when expressed as a fraction of B_2_ (Figure 3), heart rate during end-recovery after exposure to MIH was similar to B_2_ on both days (*p* > 0.765). Heart rate was similar to B_2_ during all time points in the control group on the initial and final day when absolute (Figure 2) or standardized (Figure 3) data was compared (*p* > 0.703).

#### 3.3.2 Daily Mild Intermittent Hypoxia

Heart rate was not different during B_2_ on the final day compared to the initial day in the experimental group (Figures 2, 3) (*p* = 0.821). In the experimental group, the heart rate response to hypoxia during the first or last hypoxic episode was similar on the final day compared to the initial day (*p* ≥ 0.46). Heart rate during end-recovery was similar on the final day compared to the initial day when both the absolute (*p* = 0.859) and standardized (*p* ≥ 0.995) data was compared (Figures 2, 3). Heart rate during B_2_ and recovery were similar on the initial and final day in the control group (Figures 2, 3) (*p* ≥ 0.143).

### 3.4 Systolic Blood Pressure

#### 3.4.1 Acute Mild Intermittent Hypoxia

As expected, exposure to each episode of hypoxia led to an increase in systolic blood pressure compared to B_2_ (Figures 2, 3—NB symbols indicating statistical significance not shown for this expected increase) (*p* ≤ 0.026 for all comparisons). In addition, the systolic blood pressure response to hypoxia gradually increased from first to the last episode of the MIH protocol on both the initial (*d* = 0.92) and final day (*d* = 1.28) (Figures 2, 3) (*p* < 0.001 for both the initial and final day). This gradual increase was evident when the Δ in blood pressure was standardized to Δ in the partial pressure of end-tidal oxygen (Figure 4B) (*p* < 0.001). The systolic blood pressure response and ventilatory response to hypoxia were correlated (R^2^ = 0.82, *p* < 0.001).

The results also showed that systolic blood pressure during the end recovery period (i.e., following exposure to hypoxia) was greater compared to B_2_ on both the initial (*d* = 1.13) and final day (*d* = 1.59). These findings were evident when both absolute (Figure 2) (*p* < 0.001) and standardized (Figure 3) (*p* < 0.001) data was used to make comparisons. In contrast, these modifications were not evident in the control group (Figures 2, 3) (*p* > 0.713 for all comparisons, day 1 *d* = 0.41, day 15 *d* = 0.10). Consequently, systolic blood pressure during end recovery on the initial and final day was greater in the experimental group compared to control when the standardized data was compared (Figure 3) (*p* ≤ 0.001).

#### 3.4.2 Daily Mild Intermittent Hypoxia

In the experimental group, B_2_ systolic blood pressure was significantly higher on the initial day compared to the final day (*p* ≤ 0.001, *d* = 1.01). This difference was also evident during the hypoxic episodes (1-2,7-8,11-12), normoxic recovery periods (1–2, 7–8, 11), and end-recovery (ER1 and ER2) periods in the protocol (Figure 2) (*p* < 0.005 for all comparisons—NB symbols indicating statistical significance for every time point not shown). The magnitude of the progressive increase in systolic blood pressure, from the first to the last hypoxic episode of the MIH protocol, was similar on the initial and final day (Figure 3) (*p* ≥ 0.401). This similarity was also evident when the systolic blood pressure response to hypoxia (Δ in blood pressure divided by Δ in the end-tidal partial pressure of oxygen) during the first episode or last episode were compared on the initial and final day (Figure 4B) (*p* = 0.843). Likewise, the magnitude of the increase in systolic blood pressure during the end-recovery period compared to B_2_ was similar on the initial and final day of the protocol (*p* = 0.450). No modifications were evident in the control group when data collected on the initial and final day of the protocol were compared (Figures 2, 3) (*p* > 0.333 for all comparisons, B_2_ *d =* 0.11; episode 1 *d =* 0.36; episode 12 *d =* 0.02; end-recovery *d =* 0.20).

### 3.5 Blood Pressure Variability

#### 3.5.1 Acute Mild Intermittent Hypoxia

**FIGURE 5:**
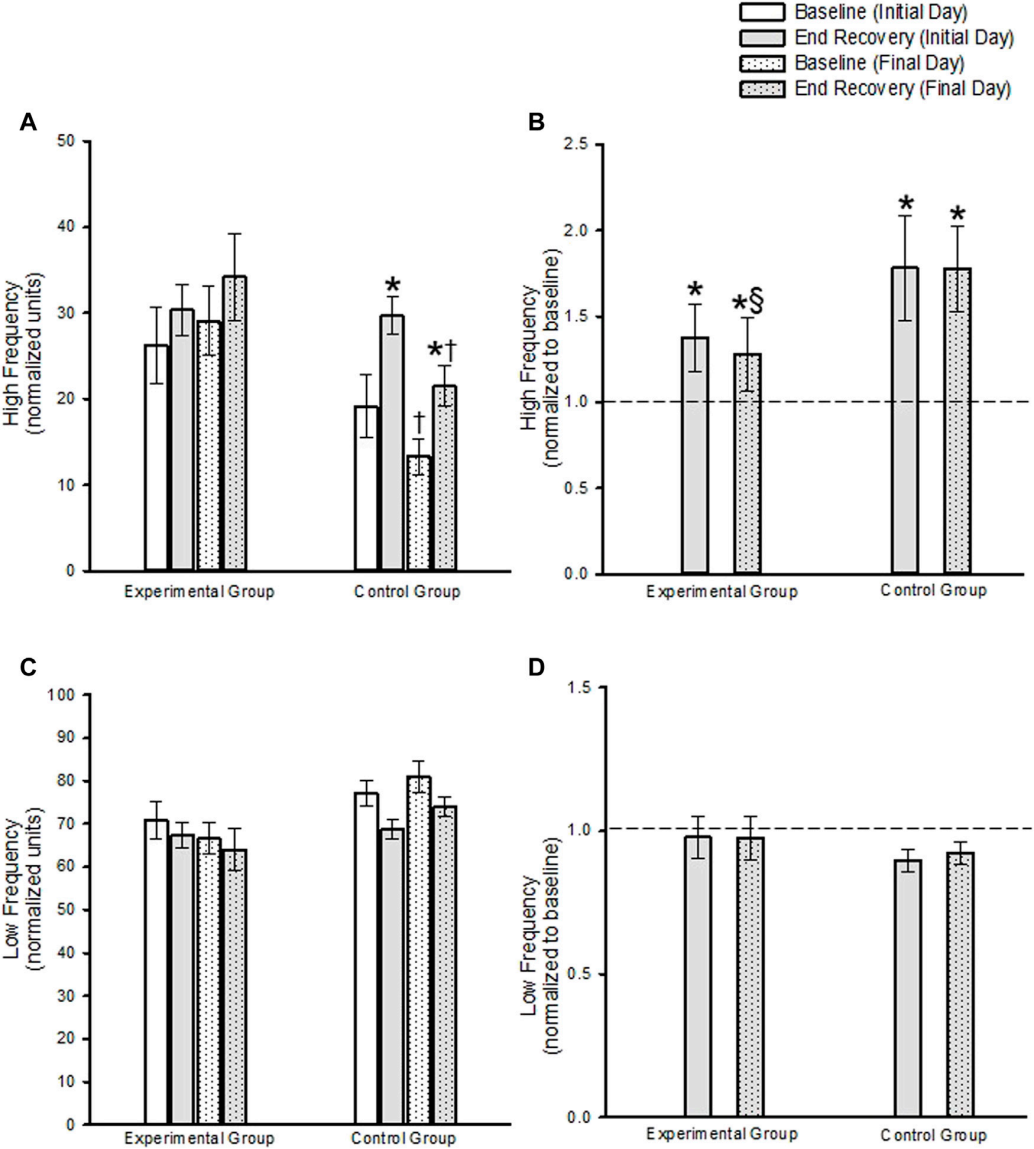
**(A)** Absolute and **(B)** standardized measures (standardized to baseline on a given day) of the high frequency component of blood pressure variability during baseline and end-recovery. Note in **(B)** that the dashed line represents baseline. **(C)** Absolute and **(D)** standardized measures of the low frequency component of blood pressure variability during baseline and end-recovery. Note in **(D)** that the dashed line represents baseline. * = significantly different compared to B_2_. ^‡^ = Significantly different than episode 1 on both days. ^§^ = Significantly different than controls at the same respective time. Significance set at *p* < 0.05. Data presented as mean ± Standard Error.

In the experimental group, HF measures of blood pressure variability during the end-recovery period following MIH were similar to B_2_ measures on the initial and final day of the protocol (Figure 5A) (*p* ≥ 0.293). When the findings were standardized to B_2_, the results indicated that increases in high frequency measures were evident during end-recovery compared to B_2_ (Figure 5B) (*p* < 0.001). In the control group high frequency measures of blood pressure variability increased during the end-recovery period compared to B_2_ on both the initial and final day when both absolute (Figure 5A, *p* ≥ 0.020) and standardized measures (Figure 5B, *p* < 0.001) were compared.

B_2_ measures tended to be lower in the control group compared to the experimental group (*p* < 0.058). Thus, the comparison of high frequency blood pressure variability measures during end-recovery on a given day, between the experimental and control group, were determined using standardized data. These results showed that after standardizing to baseline values, high frequency blood pressure variability measures during end-recovery were similar in the control group compared to the experimental group on the initial day (Figure 5B, *p* = 0.973). In contrast, the high frequency blood pressure measures were greater in the control group compared to the experimental group on the final day (Figure 5B, *p* = 0.030).

In the experimental group LF blood pressure variability measures during end recovery were not different from B_2_ on the initial and final day of the protocol, when absolute (Figure 5C, *p* = 0.528) or standardized data (Figure 5D, *p* = 0.969) were compared. In the control group, a decrease in low frequency blood pressure variability during the end-recovery period on the initial day approached significance (Figure 5C, *p* = 0.051) compared to B_2,_ but was not significant on the final day (Figure 5C, *p* = 0.112) when absolute measures were compared. When the data was standardized to baseline, the decrease in low frequency blood pressure variability during end-recovery compared to B_2_ approached significance on the initial and final day (Figure 5D, *p* = 0.062 and *p* ≤ 0.058, respectively).

B_2_ measures on the initial day and final day tended to be greater in the control group compared to the experimental group (*p* = 0.071). Thus, the comparison of low frequency blood pressure variability measures during end-recovery on a given day, between the experimental and control group, were determined using standardized data. These results showed that the decrease in low frequency blood pressure variability measures during end-recovery was similar in the control group compared to the experimental group on both the initial and final day (Figure 5D, *p* = 0.477).

#### 3.5.2 Daily Mild Intermittent Hypoxia

High frequency measures of blood pressure variability during B_2_ and end-recovery were similar on the final day compared to the initial day in the experimental group (Figure 5A, *p* = 0.323). This was also the case when the standardized data was compared (Figure 5B, *p* = 0.574). In contrast, high frequency measures were lower during B_2_ and end-recovery on the final day compared to the initial day in the control group (Figure 5A, *p* ≤ 0.014. Following standardization of the data, high frequency blood pressure variability measures during end-recovery was similar on the final day compared to the initial day (Figure 5B, *p* = 0.980).

Low frequency measures of blood pressure variability were similar on the initial and final days at baseline or end-recovery in the experimental group when absolute (Figure 5C; B_2_, *p* = 0.654; end-recovery, *p* = 0.459) or standardized data (Figure 5D, *p* = 0.387) was compared. Likewise, low frequency measures of blood pressure variability were similar on the initial and final days at baseline or end-recovery in the control group when the absolute (Figure 5C B_2_, *p* = 0.585; end-recovery, *p* = 0.388) or standardized data (Figure 5D, *p* = 0.733) were compared.

No differences in the LFα index, a surrogate for baroreflex sensitivity, were found in the experimental group (9.54 ± 1.19 vs. 8.04 ± 1.05, *p* = 0.105) or the control group (9.17 ± 1.64 vs. 9.55 ± 0.85, *p* = 0.842). Additionally, no differences between groups were found on day 1 (*p* = 0.854) or day 15 (*p* = 0.337).

## 4 Discussion

Our findings showed that an acute exposure to MIH initiated progressive augmentation of the hypoxic ventilatory response and long-term facilitation of ventilation in a group of OSA participants with hypertension, which confirms and adds to our previous findings that revealed a similar response in normotensive OSA participants. However, the magnitude of progressive augmentation and long-term facilitation of ventilation was not enhanced following daily exposure to MIH. We also showed that an acute exposure to MIH induces a progressive increase in the systolic blood pressure response to hypoxia from the first to the last episode of an MIH protocol. Moreover, we showed that following MIH on a given day, exposure to this stimulus also led to a sustained increase in blood pressure. Our results also revealed that despite a sustained increase in blood pressure following acute exposure to MIH, daily exposure to MIH resulted in a reduction in resting blood pressure. The reduction in resting blood pressure, coupled with a similar hypoxic blood pressure response, resulted in diminished absolute blood pressure measures both during and following exposure to MIH on the final day.

### 4.1 Hypoxic Ventilatory Response and Systolic Blood Pressure Response to Hypoxia

Findings from the present investigation showed that the ventilatory response to hypoxia was greater during the last compared to the first hypoxic episode of the MIH protocol. We have observed this phenomenon in previous investigations (Lee et al., 2009; Gerst et al., 2011; Syed et al., 2013). We also showed that the magnitude of the hypoxic ventilatory response was not enhanced following daily exposure to MIH. This finding is in contrast to our previous results, and the results from other laboratories, which showed that the ventilatory response to hypoxia or hypercapnia was enhanced following daily exposure to MIH (Bernardi et al., 2001; Foster et al., 2005; Haider et al., 2009; Gerst et al., 2011). This discrepancy might be related to dissimilarities in the MIH protocol that was employed. For example, in our previous investigation 4-min episodes (Gerst et al., 2011) were used compared to the 2-min episodes employed in the present study. Moreover, the dose of hypoxia used in previous studies from other laboratories were more severe in regard to hypoxic intensity (Bernardi et al., 2001; Haider et al., 2009) and episode duration (Bernardi et al., 2001; Foster et al., 2005; Haider et al., 2009; Gerst et al., 2011). This suggestion is supported by animal studies (Nichols et al., 2012) which reported that progressive augmentation of the ventilatory response to hypoxia was dependent on dose severity.

Similar to the results obtained from measures of minute ventilation, we showed that an acute exposure to MIH resulted in progressive augmentation of the systolic blood pressure response to hypoxia. The progressive increase in systolic blood pressure could be the consequence of enhanced sympathetic nervous system activity. Unfortunately, the length of the hypoxic episodes that we employed in the present study did not allow us to obtain non-invasive measures of sympathetic nervous system activity. Nonetheless, some studies have shown increases in muscle sympathetic nerve activity during exposure to various hypoxia protocols during wakefulness (Xie et al., 2001; Cutler et al., 2004b; Tamisier et al., 2004; Querido et al., 2010; Jouett et. al., 2017).

We found that the magnitude of the progressive increase in minute ventilation and systolic blood pressure were correlated. Thus, a similar mechanism could be responsible for the coupling of the progressive enhancement of minute ventilation and systolic blood pressure during exposure to MIH. Previous work completed in both humans and animals suggests that input from the carotid bodies (Duffin, 2007; Peng and Prabhakar, 2003) might be responsible for the progressive enhancement of both respiratory and sympathetic medullary neuronal activity (Mitchell and Johnson, 2003). Indeed, progressive augmentation of carotid sensory nerve activity has been recorded in rats in response to exposure to hypoxia (Peng and Prabhakar, 2003; Peng et al., 2011; Roy et al., 2017). The augmented carotid response to hypoxia was abolished when rats were treated with a potent antioxidant (Peng and Prabhakar, 2003) or antihypertensive medications (Roy et al., 2017). In support of these results obtained in animals, we showed that progressive augmentation of the ventilatory response to hypoxia was eliminated following administration of antioxidants in humans with OSA [see Figure 2 in (Lee et al., 2009)].

Although the systolic blood pressure response to hypoxia increased from the first to the last hypoxic exposure on a given day, the blood pressure response to hypoxia was similar on the final day compared to the initial day. This lack of an enhanced response is similar to the observation made for minute ventilation. If the carotid bodies are responsible for induction of progressive augmentation of both blood pressure and minute ventilation, our results suggest that daily exposure to our protocol did not modify the phenomenon beyond that induced by acute exposure to the stimulus. In contrast, others have reported dissimilarities in the minute ventilation and blood pressure response to daily exposure to MIH (Bernardi et al., 2001; Foster et al., 2005; Chacaroun et al., 2016). More specifically, previous results have shown enhancement in minute ventilation following daily exposure to MIH without an accompanying enhancement of the blood pressure response. This finding may suggest that despite progressive enhancement of peripheral chemoreceptor sensitivity following daily exposure to MIH the influence of afferent feedback on respiratory and sympathetic neuronal discharge may vary between the neuronal groups.

### 4.2 Sustained Increases in Minute Ventilation and Systolic Blood Pressure following Mild Intermittent Hypoxia

Our findings showed that following acute exposure to MIH sustained increases in minute ventilation and systolic blood pressure were evident. The sustained increase in blood pressure could be indicative of a sustained increase in sympathetic nervous system activity. Indeed, other published findings have reported that muscle sympathetic nervous system activity and blood pressure is elevated following exposure to intermittent hypoxia in humans (Jouett et al., 2017; Stuckless et al., 2020). As speculated above, the sustained elevation in minute ventilation and blood pressure might originate from the carotid bodies, since long term facilitation of carotid sensory nerve activity has also been documented in rats (Peng and Prabhakar, 2003; Peng et al., 2011). However, sustained increases in blood pressure have not been consistently observed following acute exposure to MIH (Cutler et al., 2004a; Cutler et al., 2004b; Vermeulen et al., 2020), suggesting that other mechanisms may have a role in sustained increases (or lack thereof) in blood pressure.

Although the magnitude of the sustained increase in minute ventilation and blood pressure following acute MIH was similar on the initial and final day of the protocol, our results revealed that resting blood pressure on the final day, prior to exposure to MIH, was reduced while minute ventilation remained unaltered. The combination of a reduction in resting blood pressure coupled to a similar hypoxic blood pressure response resulted in diminished absolute blood pressure measures both during and following exposure to MIH on the final day. Thus, in addition to the mechanism responsible for a similar minute ventilation and blood pressure response during and following acute exposure to MIH (i.e., carotid bodies), additional mechanisms may manifest following repeated exposure to MIH that leads to uncoupling of resting minute ventilation and blood pressure measures. The reduction in blood pressure that was observed may have been due to a reduction in baseline sympathetic nervous system activity (i.e., normalized low-frequency blood pressure variability) (Panza et al., 2022) and/or baseline modifications in microvascular function. Our measures of blood pressure variability did not provide strong evidence that reductions in sympathetic nervous system activity contributed to the observed reductions in blood pressure. Specifically, decreases in sympathetic nervous system activity during the second baseline period (i.e., when the partial pressure of carbon dioxide was sustained slightly above baseline measures) and following exposure to intermittent hypoxia on the last day of the protocol were not evident. In contrast, based on our measures of blood pressure variability, parasympathetic nervous system activity may have increased following daily repeated exposure to MIH. However, given that a similar increase was evident in the control group we do not have strong evidence that modifications in parasympathetic nervous system activity played a role in the overall reduction in blood pressure observed throughout the MIH protocol.

Thus, uncoupling of sympathetic nervous system activity and blood pressure may have occurred. Uncoupling of muscle sympathetic nervous system activity and blood pressure following exposure to hypoxia has been reported previously (Cutler et al., 2004a; Cutler et al., 2004b; Lusina et al., 2006; Querido et al., 2010). Local vasodilatory effects of systemic hypoxia could override vasoconstrictor influences from the increased sympathetic outflow resulting in changes in vascular resistance that are unexpected based on sympathetic nervous system activity (Puri et al., 2021). Indeed, Xie et al. (2001) reported sustained increases in muscle sympathetic nerve activity following hypoxia without significant modifications in blood pressure or leg blood flow, suggesting that the amount of sympathetic activation was insufficient to raise total peripheral resistance or that the short exposures to hypoxia may not elicit pro-hypertensive mechanisms. Conversely, local vasodilators, such as adenosine and nitric oxide (Costa and Biaggioni, 1998; Sato et al., 2005), could explain the lack of increase in blood pressure despite increases in sympathetic activity (Xie et al., 2001). For example, significant increases in nitric oxide have been reported following intermittent hypoxia in hypertensive patients with a concurrent reduction in blood pressure (Lyamina et al., 2011; Muangritdech et al., 2020) supporting the role of peripheral mechanisms in reducing blood pressure.

Independent of the mechanisms, our previous and present results indicate that acute exposure to MIH can lead to a progressive increase in the minute ventilation and blood pressure response to hypoxia, that is ultimately sustained following exposure to MIH. On the other hand, respiratory, autonomic and cardiovascular outcome measures may become uncoupled following daily exposure to MIH. More specifically, a reduction in resting blood pressure may become evident after days of exposure, despite the maintenance or increase of minute ventilation and possibly sympathetic nervous system activity.

## 5 Conclusion and Translational Significance

Over the years there has been a great deal of interest in the impact that intermittent hypoxia has in initiating respiratory and autonomic plasticity. Most recently, the focus has been on determining if MIH can be used as a therapeutic modality to reverse the course of a number of detrimental outcome measures. Our findings have shown that daily exposure to MIH elicit complex outcomes in minute ventilation and blood pressure. Sustained increases in minute ventilation and blood pressure are evident for a period of time after acute exposure. In contrast, blood pressure is reduced after daily exposure while minute ventilation remains unchanged. The reduced blood pressure after daily exposure might be indicative of reductions in baseline sympathetic nervous system activity, or based on our findings, could be indicative of modifications in the microvasculature in the presence of unchanging sympathetic nervous system activity. Our findings not only have implications for the use of MIH as a therapeutic modality but may also provide insight into outcomes for individuals suffering from OSA. It may be that acute exposure to MIH leads to a temporary increase in blood pressure but that long term exposure to MIH may induce beneficial outcomes which mitigates detrimental outcomes induced by other hallmarks of OSA.

## Data Availability

The raw data supporting the conclusion of this article will be made available by the authors, without undue reservation.
